# Comparative study between *Helicobacter pylori* and host human genetics in the Dominican Republic

**DOI:** 10.1186/s12862-019-1526-9

**Published:** 2019-11-01

**Authors:** Takaaki Ono, Modesto Cruz, José A. Jiménez Abreu, Hiroyuki Nagashima, Phawinee Subsomwong, Celso Hosking, Seiji Shiota, Rumiko Suzuki, Yoshio Yamaoka

**Affiliations:** 1 Department of Environmental and Preventive Medicine, Faculty of Medicine, Oita University, Yufu, Oita Japan; 2 Criminal Investigation Laboratory, Oita Prefectural Police H.Q, Oita, Japan; 3 Institute of Microbiology and Parasitology, Faculty of Science, Autonomous University of Santo Domingo, Santo Domingo, Dominican Republic; 4 Department of Biomedical Research, National Institute of Medicine and Diagnostic Imaging, Santo Domingo, Dominican Republic; 5 Dominican-Japanese Digestive Disease Center, Dr Luis E. Aybar Health and Hygiene City, Santo Domingo, Dominican Republic; 6 Department of Gastroenterology, Hokkaido Cancer Center, Sapporo, Hokkaido Japan; 7 Department of Medicine, Gastroenterology and Hepatology Section, Baylor College of Medicine, Houston, TX USA

**Keywords:** *Helicobacter pylori*, Population structure, Dominican Republic, Phylogeography, Genetic diversity, Human mitochondrial DNA, Human Y-chromosome DNA

## Abstract

**Background:**

*Helicobacter pylori*, a bacterium that infects the human stomach, has high genetic diversity. Because its evolution is parallel to human, *H. pylori* is used as a tool to trace human migration. However, there are few studies about the relationship between phylogeography of *H. pylori* and its host human.

**Methods:**

We examined both *H. pylori* DNA and the host mitochondrial DNA and Y-chromosome DNA obtained from a total 119 patients in the Dominican Republic, where human demography consists of various ancestries. DNA extracted from cultured *H. pylori* were analyzed by multi locus sequence typing. Mitochondrial DNA and Y-chromosome DNA were evaluated by haplogroup analyses.

**Results:**

*H. pylori* strains were divided into 2 populations; 68 strains with African group (hpAfrica1) and 51 strains with European group (hpEurope). In Y-chromosomal haplogroup, European origin was dominant, whereas African origin was dominant both in *H. pylori* and in mtDNA haplogroup. These results supported the hypothesis that mother-to-child infection is predominant in *H. pylori* infection. The Amerindian type of mtDNA haplogroup was observed in 11.8% of the patients; however, Amerindian type (hspAmerind) of *H. pylori* was not observed. Although subpopulation type of most hpAfrica1 strains in Central America and South America were hybrid (hspWAfrica/hpEurope), most Dominican Republic hpAfrica1 strains were similar to those of African continent.

**Conclusions:**

Genetic features of *H. pylori*, mtDNA, and Y haplogroups reflect the history of colonial migration and slave trade in the Dominican Republic. Discrepancy between *H. pylori* and the host human genotypes support the hypothesis that adaptability of hspAmerind *H. pylori* strains are weaker than hpEurope strains. *H. pylori* strains in the Dominican Republic seem to contain larger proportion of African ancestry compared to other American continent strains.

## Background

*Helicobacter pylori* (*H. pylori*) is a spiral, gram-negative human pathogen and is a major cause of peptic ulcer disease and gastric cancer. We previously reported that *H. pylori* had already coexisted in the human stomach about 60 thousand years ago when the anatomically modern human radiated from the African continent [1, 2]. Currently about 50% of world population is infected with this bacterium [3].

Since *H. pylori* frequently undergoes recombination among unrelated strains, its genetic diversity is higher compared with other bacteria, and is about 50 times higher than that of human [4–6]. This large genetic diversity of *H. pylori* helps us to predict host human migration across the world [7, 8]. In recent years, geographical information of *H. pylori* has been attempted to apply to forensic science for detailed survey of unidentified corpses [9, 10]. Furthermore, a comparative study of *H. pylori* and human DNA in Ladakh of India demonstrated that genetic diversity of *H. pylori* was more informative than human mitochondrial DNA (mtDNA) [11].

*H. pylori* infection occurs mainly vertically, as well as horizontally [12, 13]. In horizontal infection, the risk factor is sanitary conditions such as undeveloped water supply and sewerage and residential environment. Most of the infection is established during infancy until 2 years old when immunity is not sufficiently developed [14]. Vertical infection occurs during this period between intimate family members like a mother and the child [14, 15]. Because vertical infection is dominant, the evolution of *H. pylori* is basically parallel to the host human [16]. As for the Dominican Republic, the World Health Organization and UNICEF have reported that the percentage of improved sanitation facilities in the Dominican Republic in 2015 was 84% [17]. Because the sanitary conditions are not completely improved, *H. pylori* infection in the Dominican Republic can be both vertical and horizontal. However, when multiple strains of different lineage were infected in a patient, the more adaptive strain may overcome the existent strain or hybridize with each other to form a mosaic strain [18–21].

A previous study hypothesized that adaptability of *H. pylori* strains which had been originally infected to American aborigines might be lower than European strains brought by colonial settlers after Columbus’s landing [22–25].

A conventional phylogenetic and population analysis of *H. pylori* are based on Multi Locus Sequence Typing (MLST), which sequences seven housekeeping genes (*atpA*, *efp*, *mutY*, *ppa*, *trpC*, *ureI*, *yphC*) for typing [8]. According to population analyses based on MLST, *H. pylori* in the world are classified into 7 population groups: hpEurope, hpEastAsia, hpAsia2, hpAfrica1, hpAfrica2, hpNEAfrica, and hpSaful [1, 16, 21, 26]. The hpEastAsia is subdivided into three subpopulations: hspMaori, hspAmerind, and hspEAsia. The hspMaori is commonly observed in Polynesia, Melanesia and Taiwanese aborigines, hspAmerind is observed in American aborigines, and hspEAsia is commonly observed in East Asia.

Human population was conventionally studied using mtDNA haplogroup and Y-chromosomal haplogroup (Y-haplogroup) as lineage markers [27–30]. The mtDNA is 16,569 bp long and contains two highly polymorphic segments (hypervariable regions, HV1 and HV2) that are located in control region (D-loop region), which does not encode genes. The mtDNA is inherited maternally without recombination and it has been studied for long time. Substantial databases of mtDNA are developed around the world. Y-chromosome DNA is inherited paternally and undergoes recombination only in a limited region such as pseudoautosomal region (PAR).

Although the adverse effect of *H. pylori* on the digestive system of human hosts is mainly caused by pathogenic genes such as *cagA*, it is hypothesized that the population types to which the infected bacterium belongs may also affect clinical symptoms [31, 32]. Recent studies in Colombia show a mismatch of host (human) ancestry versus bacterial (*H. pylori*) ancestry can lead more severe gastric mucosal damages, thus coevolution likely modulated disease risk [33].

The Dominican Republic is located on the east side of Hispaniola Island in the Caribbean. This island accommodated the first permanent European settlement founded by Christopher Columbus. Our recent study showed the overall prevalence of *H. pylori* infection was 58.9% [34]. The ethnics in the Dominican Republic consists of 16% of European, 11% of African, and 73% of mixed race [35]. It is estimated that 2 to 7 million indigenous people had lived in the Caribbean before the Columbus’s landing in 1492 [36]. Although pure descendants of American aborigines in the Dominican Republic were lost after Columbus and successive settlement of African slaves, the Dominican Republic people are expected to have three ancestral components. In this study, we evaluated correspondence between *H. pylori* and human genetics in the Dominican Republic population.

## Methods

### Study population and DNA extraction

Biopsy specimens from gastric mucosa were taken from 258 dyspeptic patients (158 in 2011 and 100 in 2016; 86 males and 172 females; age range, 17–91 years; mean age, 46.2 ± 15.8 years) who underwent endoscopy examination at the Digestive Disease Center, Dr. Luis E. Aybar Health and Hygiene City, Santo Domingo, Dominican Republic. Of these patients, 219 had chronic gastritis, 38 had peptic ulcer diseases, and one had gastric cancer. For *H. pylori* culture, antral biopsy specimens were homogenized and inoculated onto antibiotic selection plates, and then subcultured on Mueller Hinton II Agar medium (Becton Dickinson, Sparks, MD) supplemented with 7% horse blood without antibiotics. The plates were incubated up to 10 days at 37 °C under microaerophilic conditions (10% O2, 5% CO2, and 85% N2). *H. pylori* isolates were identified based on colony morphology; Gram staining results; and oxidase, catalase, and urease reactions. Isolated strains were stored at − 80 °C in *Brucella* broth (Becton Dickinson, Sparks, MD) containing 10% dimethyl sulfoxide and 10% horse serum. Bacterial DNA was extracted using a commercially available kit (QIAGEN Inc., Valencia, CA, USA). Eventually, 64 strains were cultured from 158 patients in 2011, and 56 strains were cultured from 100 patients in 2016, thus in total 120 *H. pylori* strains could be obtained. Human DNA was also extracted from biopsies by the same QIAGEN kit. Human DNA of one sample in 2011 had already used up in our previous study [37], therefore we excluded a *H. pylori* strain that does not have corresponding human DNA and used the rest of 119 strains of *H. pylori* and host human DNA in this study. The ethnicity of the 119 patients based on self-assessment at the time of medical examination was 113 multiracial (31 males, 82 females) and 6 African (3 males, 3 females). Written informed consent was obtained from the all participants, and the protocol was approved by the ethics committees of Dr. Luis E. Aybar Health and Hygiene City, the Institute of Microbiology and Parasitology, IMPA, Autonomous University of Santo Domingo, UASD, in the Dominican Republic and Oita University Faculty of Medicine, Japan.

### Analysis of the host human DNA

The control region of mtDNA sequencing was performed by PCR-based sequencing as described previously [38]. Primers for PCR amplification and direct sequencing are shown in Additional file 1: Table S1. The sequences were aligned and compared with the revised Cambridge Reference Sequence (rCRS) [39, 40] using MEGA software (version 7.0) [41]. After the consensus sequence of each individual was obtained, polymorphisms were examined. The mtDNA haplogroup was determined by Haplogrep software (version 2.0, https://haplogrep.uibk.ac.at/) [42] or by direct comparison with PhyloTree 17 data [43]. For individuals that were difficult to judge mtDNA haplogroup by the control region, single nucleotide polymorphism (SNP) of the coding region were also examined as described previously [44].

On 34 male samples, 17 loci of Y-chromosomal short tandem repeats (Y-STR) were determined by AmpFlSTR Yfiler Kit (Applied Biosystems, Foster city, CA) according to the manufacturer’s instructions. Y-haplogroup was predicted by Haplogroup Predictor (http://www.hprg.com/hapest5/). Based on the results of Y-haplogroup estimated from Y-STR, 12 types of Y-SNP markers (M168, M145, P170, M201, M170, P209, M213, M9, M45, M207, M198, M343) were selected referring to the previous method, and all SNPs were determined by SNaPshot method as described previously [45]. Primers are shown in Additional files 2 and 3: Tables S2 and S3.

### Analysis of *H. pylori* population structure

Seven housekeeping genes (*atpA*, *efp*, *mutY*, *ppa*, *trpC*, *ureI*, *yphC*) of MLST were determined by PCR-based sequencing as described previously [5]. Primers for PCR amplification and direct sequencing are shown in Additional file 4: Table S4. For construction of phylogenetic tree, 1293 MLST sequences of global strains were obtained from the previous studies: 229, 67, 113, 544, 92, 50, 128, 39, and 31 strains from hpAfrica1, hpAfrica2, hpNEAfrica, hpEurope, hpAsia2, hpSahul, hspEAsia, hspMaori, and hspAmerind, respectively (Additional file 5: Table S5) [1, 2, 11, 16, 18, 21, 22,31, 46–53]. We used MLST sequences of our Dominican Republic strains and these global strains for the successive analyses. Neighbor-joining trees were constructed by MEGA software (version 7.0) using Kimura-2 parameters model [41, 54, 55]. To investigate the population structure of the Dominican Republic strains, we executed Bayesian population assignments by STRUCTURE software (version 2. 3. 3), using “no-admixture model” and “linkage model” [56], on totally 1295 strains: the 119 Dominican Republic strains and 1176 published global reference sequences (excluding hpAfrica2 and hpSahul from the above 1293 reference strains because their groups had no relevance with the Dominican Republic strains in the phylogenetic tree). To determine the number of bacterial populations (K) within the dataset, STRUCTURE was executed by setting K from 4 to 7 (10 runs for each K) with 30,000 iterations following a burn-in period of 20,000 iterations. Further STRUCTURE analyses were performed to detect subpopulations for each hpEurope and hpAfrica1 group by setting K from 2 to 5 (10 runs for each K). The STRUCTURE runs with the highest posterior probability among 10 runs were used for subsequent analyses such as statistical test.

To investigate which strain of African continent is close to the hpAfrica1 strains in the Dominican Republic, a phylogenetic tree was constructed incorporating only strains that belonged to hpAfrica1 with probability of 100% in no-admixture model of the STRUCTURE (K = 5, 1295 strains) as pure hpAfrica1 strains (163 reference and 45 Dominican Republic). Neighbor-joining trees were constructed by MEGA software (version 7.0) using Kimura-2 parameters.

Estimates of the number of polymorphic sites, haplotype diversity and nucleotide diversity were calculated by the Arlequin software (version 3.5) [57]. Pairwise genetic distances were calculated by MEGA software (version 7.0) using Kimura-2 parameters. Statistical significance was tested using Kruskal-Wallis test, Fisher’s exact test, and Wilcoxon rank sum test implemented in the R package (version 3.4.0). A *p*-value of < 0.05 was accepted as statistically significant.

## Results

### Host human DNA

In mtDNA analysis, a total of 715 bp nucleotide sequences of HV1 (16024–16,400) and HV2 (73–407) were determined from all the 119 human DNA. There were 94 different mtDNA haplotypes and sequences of 82 individuals were all different from each other. The haplotype diversity and the nucleotide diversity of the 715 bp mtDNA control region of the 119 individuals were 0.9930 ± 0.0026 and 0.021 ± 0.01, respectively. Thus, 119 patients were not thought to have biased kinship. Of the 12 haplotypes shared by multiple individuals, 4 were shared by 2 individuals, 5 were shared by 3 individuals, 2 were shared by 4 individuals and 1 was shared by 6 individuals. These 119 individuals were divided into 3 geographical classifications by mtDNA haplogroups. 96 belonged to African type: 9 were haplogroup L0a, 12 were L1 (8 and 4 of L1b and L1c, respectively), 30 were L2 (12, 6, 9, and 3 of L2a, L2b, and L2c, respectively), 43 were L3 (11, 11, 15 and 6 of L3b, L3d, L3e, and L3f, respectively), one was L4, one was U6. 14 belonged to Amerindian type: 5 were haplogroup A2, 3 were B2, 5 were C1, one was D1. 9 belonged to European type: 2 were haplogroup H, one was HV, 3 were J1, 3 were U5 (Additional files 6 and 7: Tables S6 and S7). In Y-STR analysis, 33 haplotypes were observed among 34 males, of which 1 haplotype was shared by 2 individuals. The haplotype diversity of Y-STR was 0.9982 ± 0.0077. These 34 males were divided into 2 geographical classifications by Y-STR and Y-SNP markers: 23 were European type (4 were haplogroup I, 7 were J, 12 were R1b) and 11 were African type (all were haplogroup E) (Additional files 7 and 8: Tables S7 and S8).

### *H. pylori* population structure

MLST sequences of 7 housekeeping genes (in total 3406 bp) were obtained from the all 119 *H. pylori* strains. MLST sequences of the 119 strains were all different from each other and contained 701 polymorphic sites. No deletion or insertion was observed. The nucleotide diversity of the MLST sequences was 0.037 ± 0.017.

We constructed a phylogenetic tree based on MLST sequences of the 119 Dominican Republic strains and 1293 reference strains. The Dominican Republic strains were located in either hpEurope or hpAfrica1 sub-branches (Additional file 9: Figure S1).

**Fig. 1:**
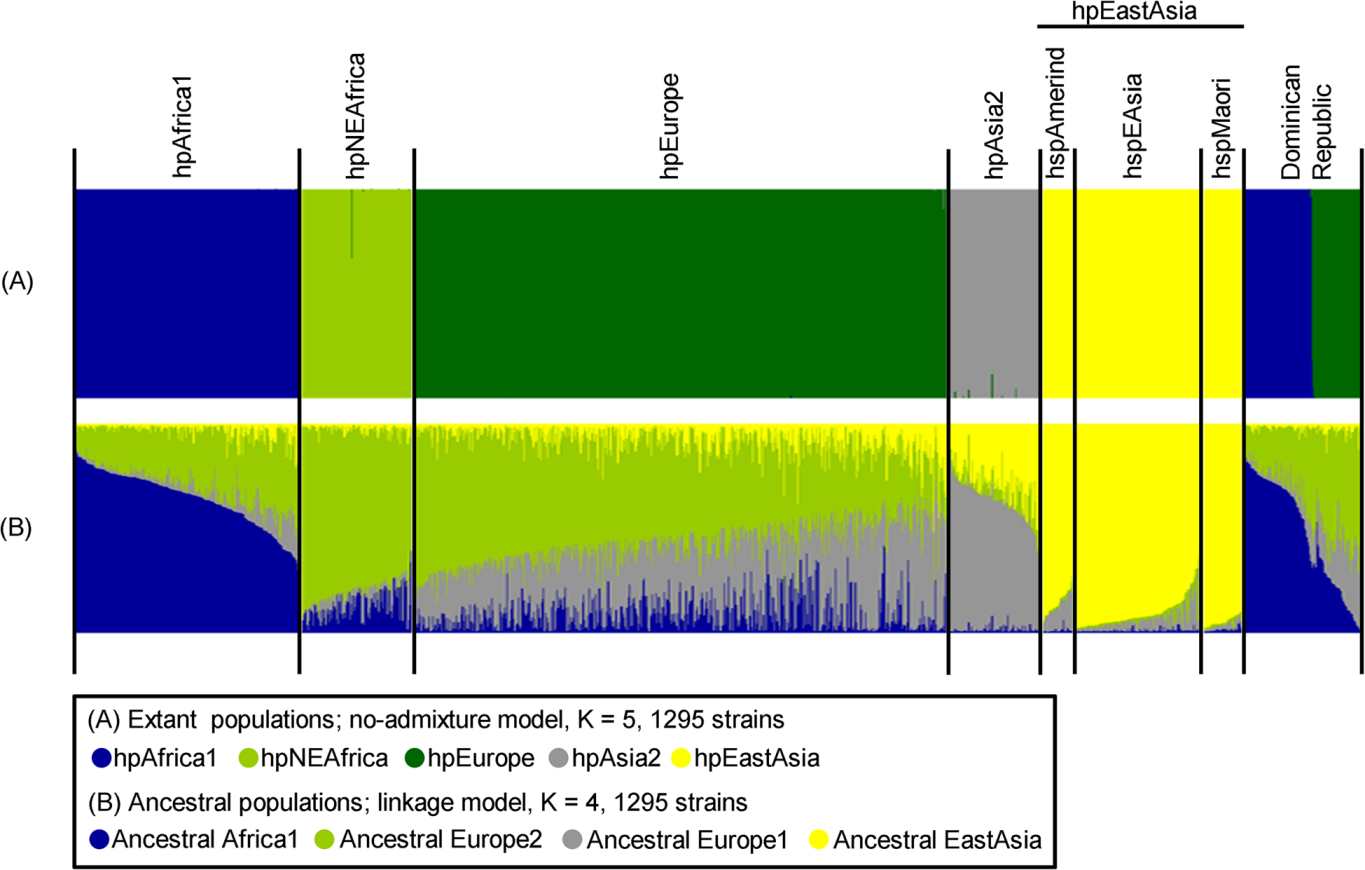
Bayesian population assignment using STRUCTURE software (version 2. 3. 3). **a** The modern population assignment of 119 Dominican Republic *H. pylori* strains with 1176 published global reference sequences by the no-admixture model (K = 5). **b** The ancestral population assignment of the Dominican Republic *H. pylori* strains with a global reference dataset by the linkage model (K = 4). Colors are coded according to the estimated population assignment (**a**) and according to the estimated amount of ancestry component (**b**). Each vertical bar represents one sample. The order of the samples is the same in each bar charts

To investigate the population structure of the Dominican Republic *H. pylori* strains, we performed population analysis using the STRUCTURE software. Figure 1a shows the result of STRUCTURE run of no-admixture model at K = 5. Under this condition, most of the global reference strains were clearly classified into the major five populations reported in the previous studies [1, 16, 26]. In the Dominican Republic, 119 strains were assigned to either hpAfrica1 (57.1%, 68/119) or hpEurope (42.9%, 51/119) (Additional file 7: Table S7). No Dominican Republic strain was classified to hpNEAfrica, hpAsia2 or hpEastAsia, and no novel population was observed. The result of STRUCTURE run of linkage model at K = 4 is very well described in Fig. 1b. Under this condition, the major four ancestral components reported in the previous studies [1, 16, 26] were observed: ancestral Africa1 (AA1), ancestral Europe 1 (AE1), ancestral Europe 2 (AE2) and ancestral EastAsia (AEA). hpEurope is known as an admixture population between AE1 and AE2 that originated from Central Asia and Northeast Africa, respectively. In the Dominican Republic, 50/51 of hpEurope strains had the higher proportion of AE2 than AE1.

**Fig. 2:**
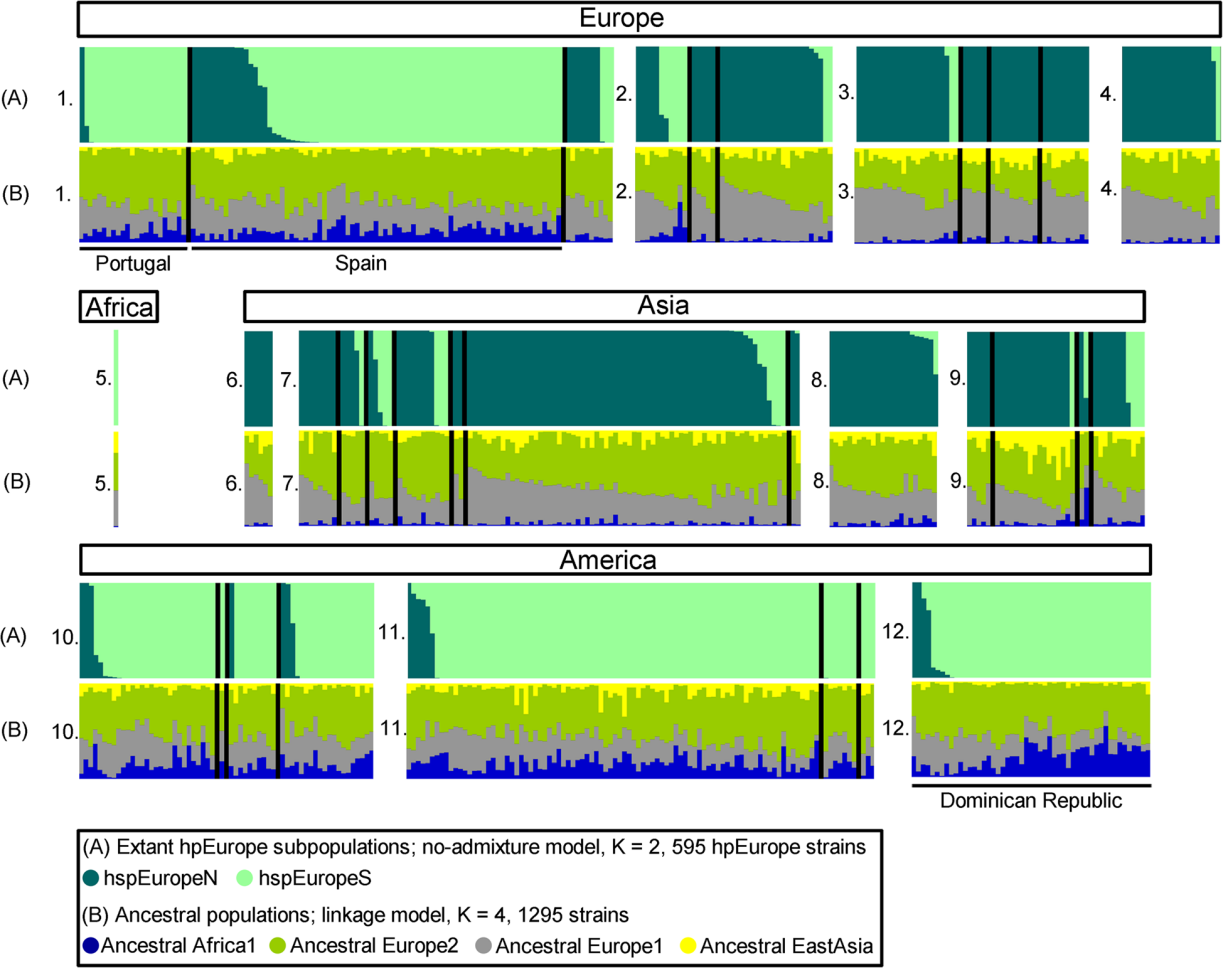
Bayesian subpopulation assignment of hpEurope strains using STRUCTURE software (version 2. 3. 3). **a** The modern subpopulation assignment of 595 hpEurope strains by the no-admixture model (K = 2). **b** The ancestral population assignment of 595 hpEurope strains by the linkage model (K = 4, 1295 strains). 1: Southern Europe (Portugal, Spain, Italy), 2: Western Europe (France, Netherlands, Germany), 3: Northern Europe (UK, Sweden, Finland, Estonia), 4: Eastern Europe (Russia), 5: Northern Africa (Algeria), 6: Central Asia (Kazakhstan), 7: Western Asia (Turkey, Lebanon, Israel, Palestine, Jordan, Iran, Arab), 8: Southern Asia (India), 9: South-Eastern Asia (Thailand, Cambodia, Vietnam, Malaysia), 10: Central America (Mexico, El Salvador, Nicaragua, Costa Rica), 11: South America (Colombia, Venezuela, Peru), 12: Caribbean (Dominican Republic). Colors are coded according to the estimated subpopulation assignment (**a**) and according to the estimated amount of ancestry component (**b**). Each vertical bar represents one sample. The order of the samples is the same in each bar charts

Next, we executed STRUCTURE no-admixture model on 51 Dominican Republic strains assigned to hpEurope and 544 global hpEurope reference strains. At K = 2, hspEuropeN and hspEuropeS were identified according to the origin of 544 reference hpEurope strains reported in the previous study [2, 16, 21] (Fig. 2). In the Dominican Republic, 4 strains were assigned to hspEuropeN (7.8%, 4/51) and 47 strains to hspEuropeS (92.2%, 47/51). In addition, we compared the ratio of ancestral component in the hspEuropeS strains dividing by geographic regions: Iberian Peninsula (*n* = 85), Central America (*n* = 53), South America (*n* = 93), Dominican Republic (*n* = 47). Statistical test showed that the Dominican Republic group had a significantly higher AA1 component than Iberian Peninsula group (Kruskal-Wallis test followed by Steel-Dwass post-hoc test, *P* < 0.001; Additional file 10: Figure S2). Likewise, the Dominican Republic group had a significantly lower AEA component than Central America group and South America group (Kruskal-Wallis test followed by Steel-Dwass post-hoc test, *P* < 0.001; Additional file 11: Figure S3). In contrast, AEA component of Iberian Peninsula group and the Dominican Republic group had no statistically significant difference.

**Fig. 3:**
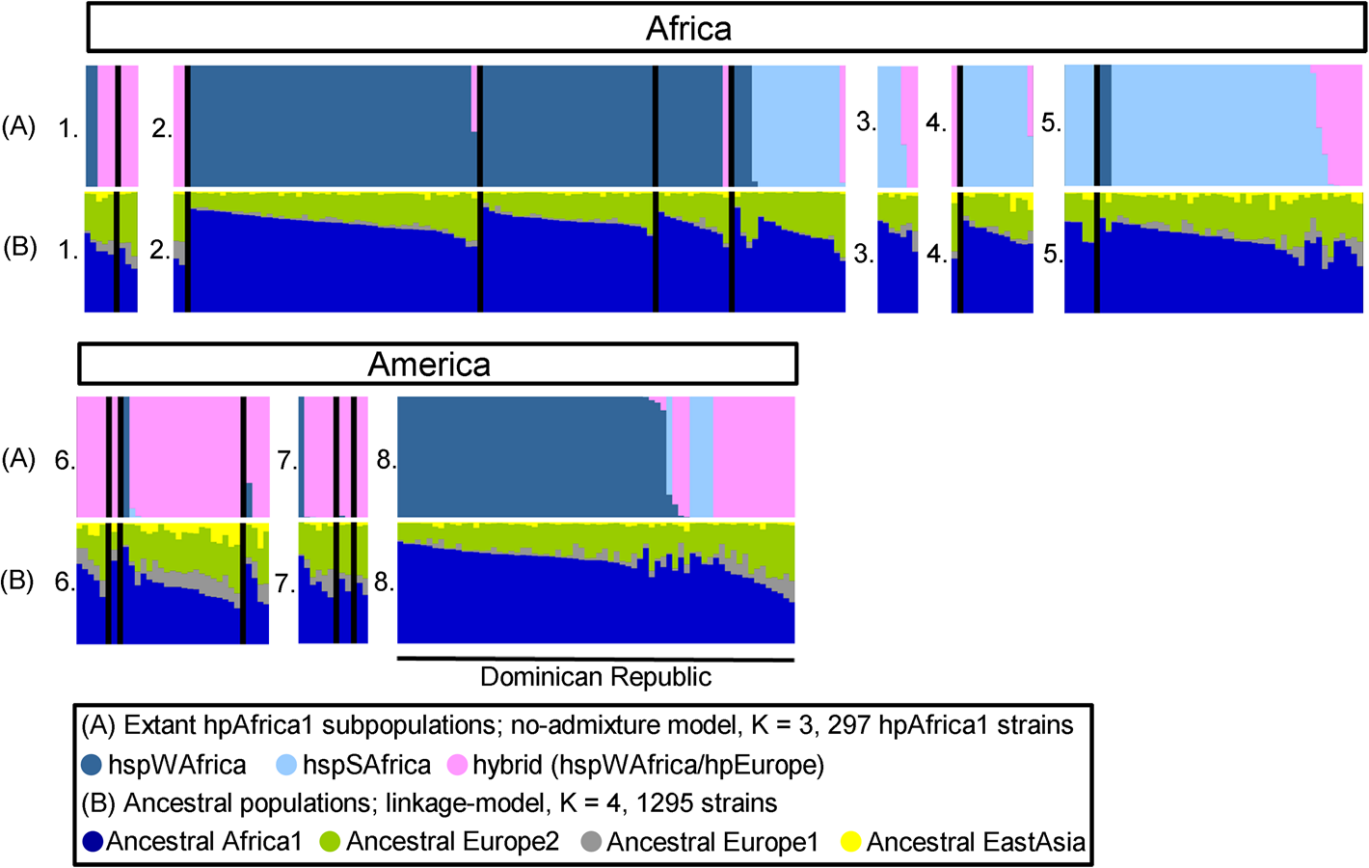
Bayesian subpopulation assignment of hpAfrica1 strains using STRUCTURE software (version 2. 3. 3). **a** The modern subpopulation assignment of 297 hpAfrica1 strains by the no-admixture model (K = 3). **b** The ancestral population assignment of 297 hpAfrica1 strains by the linkage model (K = 4, 1295 strains). 1: Northern Africa (Morocco, Algeria), 2: Western Africa (Cape Verde, Senegal, Gambia, Burkina Faso, Cameroon), 3: Middle Africa (Angola), 4: Eastern Africa (Mozambique, Madagascar), 5: Southern Africa (Namibia, South Africa), 6: Central America (Mexico, Guatemala, Nicaragua, Costa Rica), 7: South America (Colombia, Venezuela, Brazil), 8: Caribbean (Dominican Republic). Colors are coded according to the estimated subpopulation assignment (**a**) and according to the estimated amount of ancestry component (**b**). Each vertical bar represents one sample. The order of the samples is the same in each bar charts

Next, we also executed STRUCTURE no-admixture model on 68 Dominican Republic strains assigned to hpAfrica1 and 229 reference hpAfrica1 strains. At K = 2, hspWAfrica and hspSAfrica were identified according to the origin of 229 reference hpAfrica1 strains (Additional file 12: Figure S4). At K = 3, hspWAfrica was divided into two groups, but hspCAfrica subpopulation reported in the previous study was not formed [46], these three subpopulations showed different ratio of AE1 component (Fig. 3). The pink colored strains in Fig. 3 had a significantly higher ratio of AE1 component than other groups (Kruskal-Wallis test followed by Steel-Dwass post-hoc test, *P* < 0.001, respectively). Thus, these subpopulations were considered to be hspWAfrica, hspSAfrica and hybrid between hspWAfrica and hpEurope (Additional file 13: Figure S5).

**Fig. 4:**
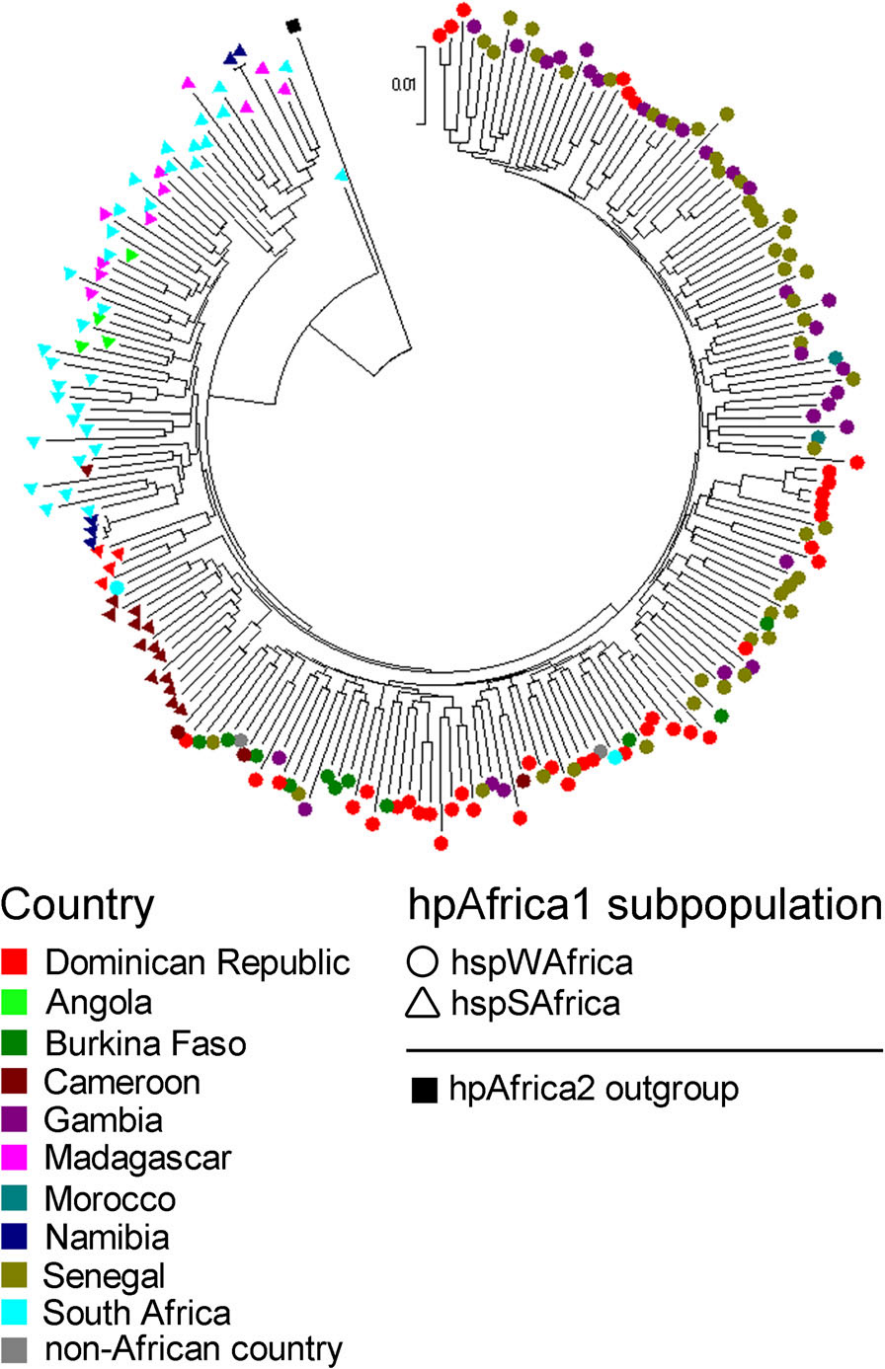
Phylogenetic relationships among hpAfrica1 strains using MEGA software (version 7.0). The samples are colored according to their geographical source and symbols (circle, triangle) indicate each subpopulation determined by STRUCTURE analysis. One hpAfrica2 strain is used as outgroup for rooting the tree

In the Dominican Republic, 68 hpAfrica1 strains were assigned to hspWAfrica (67.6%, 46/68), hspSAfrica (7.4%, 5/68) or hspWAfrica/hpEurope hybrid (25.0%, 17/68). On the contrary, 96.7% (29/30) and 90.0% (9/10) were hybrid (hspWAfrica/hpEurope) in Central America and South America, respectively. We compared the ratio of ancestral component of the hybrid (hspWAfrica/hpEurope) strains by geographic region: African continent (*n* = 24), Central America (*n* = 29), South America (*n* = 9), Dominican Republic (*n* = 17). Statistical test showed that Central America group had a significantly higher AEA component than Dominican Republic group (Kruskal-Wallis test followed by Steel-Dwass post-hoc test, P < 0.001; Additional file 14: Figure S6). At K = 4, 10 strains of classified as hybrid (hspWAfrica/hpEurope) at K = 3 formed a novel subpopulation composed of Nicaragua (*n* = 8), Guatemala (*n* = 1) and Costa Rica (*n* = 1), shown in red color in Additional file 12: Figure S4. This novel subpopulation group had a significantly higher AEA component than the other groups (Wilcoxon rank sum test, *P* = 0.003; Additional file 15: Figure S7). Next, we constructed a phylogenetic tree to investigate the origin of hpAfrica1 strains in the Dominican Republic (Fig. 4). Many of the Dominican Republic hspWAfrica strains appeared near branch of the strains from Burkina Faso, Gambia and Senegal. Four Dominican Republic hspSAfrica strains formed a cluster distant from other African strains.

### Comparison of genetic diversity by each *H. pylori* population

The number of polymorphic site and the nucleotide diversity of the 68 hpAfrica1 strains were 575 and 0.031 ± 0.014, respectively, and the number of polymorphic site and the nucleotide diversity of the 51 hpEurope strains were 535 and 0.036 ± 0.018, respectively. The genetic diversity of hpEurope population was significantly greater than hpAfrica1 population (*P* < 0.001, Wilcoxon rank sum test; Additional file 16: Figure S8).

### Relationship between phylogeographical classification of *H. pylori* and host human

**Fig. 5:**
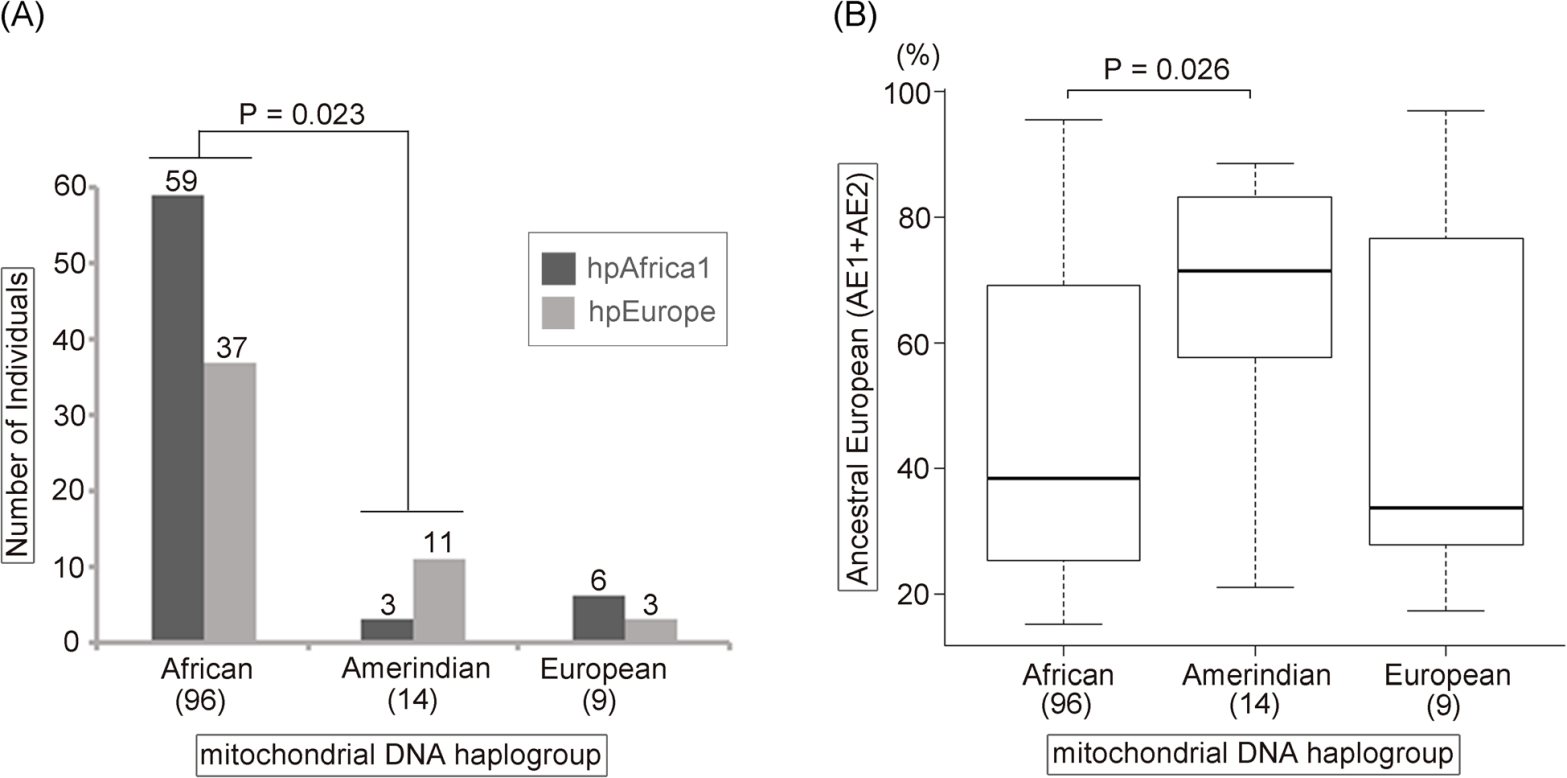
Relationship between phylogeographical classification of *H. pylori* and mitochondrial DNA haplogroup. **a** Number of *H. pylori* population type in each mitochondrial DNA haplogroup. Group comparisons were performed using Fisher’s exact test followed by Bonferroni post-hoc test. **b** Box plot diagram of European ancestry components (AE1 + AE2) in each mitochondrial DNA haplogroup. Group comparisons were performed using Kruskal-Wallis test followd by Steel-Dwass post-hoc test

Figure 5a shows the number of *H. pylori* population type in each mtDNA haplogroup. In both African and European mtDNA haplogroups, hpAfrica1 *H. pylori* was predominant. In contrast, in Amerindian mtDNA haplogroup, hpEurope was predominant. The difference of *H. pylori* populations was significant between the African and the Amerindian mtDNA haplogroups (*P* = 0.023, Fisher’s exact test followed by Bonferroni post-hoc test). In addition, Fig. 5b shows the box plot diagram of the ratio of European ancestral components (AE1 + AE2) of *H. pylori* in each mtDNA haplogroup. The AE1 + AE2 ratio was significantly different between Amerindian and African mtDNA haplogroups (*P* = 0.026, Kruskal-Wallis test followed by Steel-Dwass post-hoc test).

On the contrary, no significant difference was found between *H. pylori* population and Y-haplogroup (Additional file 17: Figure S9).

### Nucleotide sequencing

Sequencing data for seven housekeeping genes of *H. pylori* and mtDNA of human DNA are available under DDBJ accession numbers LC321074- LC321906 and LC319790- LC320027, respectively.

## Discussion

### Host human DNA

In the analysis of mtDNA haplogroup, both diversity and frequency of African type were larger than that of Amerindian type and European type. Many African mtDNA haplogroups (L1, L2, L3) observed in this study were frequent in West Africa [58, 59]. Therefore, these results reflected the history that the slave trade to the Caribbean was mainly from West Africa. All the four major Amerindian mtDNA haplogroups (A, B, C, D) [60] were observed in Amerindian type mtDNA of 14 individuals. This suggests that the human population in the Dominican Republic has a trace of ancient migrants from East Asia via the Bering Strait before Columbus. The four European mtDNA haplogroups (H, HV, J1, U5) observed in 9 individuals are common in the Iberian Peninsula where the colonial settlers were originated from [61]. Thus, the result is also consistent with the history. In Y-haplogroup analysis of 34 men, three European haplogroups (I, J, R1b) were observed, which were commonly recorded in the Iberian Peninsula [62]. The African Y-haplogroup E is common in West Africa. In contrast to mtDNA haplogroup, Amerindian Y-haplogroup was not observed at all.

In this study, African haplogroup was dominant in mtDNA and European haplotype was dominant in Y chromosome. These observations were consistent with the previous studies in the Dominican Republic [63–65]. Probably African slave men and American aboriginal men could not leave many descendants because of battle, slavery, or infectious diseases brought by Europeans during the slavery era that lasted more than 3 centuries. European mtDNA haplogroup is rare probably because only a small number of women immigrated from Europe in the past. Possible reasons why Amerindian Y-haplogroup was not observed while Amerindian mtDNA was observed is a bias for the survival of Amerindian women, the smaller sample size of Y than mtDNA or a result of miscegenation between Amerindian women and European men.

### *H. pylori* population structure

We confirmed that 119 *H. pylori* strains isolated in the Dominican Republic were classified into hpEurope and hpAfrica1. Furthermore, hpEurope strains were divided into two subpopulations: hspEuropeN and hspEuropeS. Modern hpEurope strain is a hybrid of ancestral Europe 1 (AE1), which is a main component of hpAsia2, and ancestral Europe 2 (AE2), which is a main component of hpNEAfrica. Comparing the ratio of the ancestral component of reference Eurasian continent strains used in this analysis, hspEuropeS, mainly observed in Iberian Peninsula, had higher AE2 than AE1. In contrast, hspEuropeN, mainly observed in northern Europe and Asia, had higher AE1 than AE2. In addition, hspEuropeS had a relatively high component of ancestral Africa1 (AA1), while hspEuropeN had a relatively high component of ancestral EastAsia (AEA). Furthermore, the largest subpopulation among hpEurope strains in American continent was hspEuropeS. These results were consistent with the history that the origin of the colonial settlers to American Continent was Iberian Peninsula.

Interestingly, hspEuropeS strains in Central America and Dominican Republic contain significantly higher AA1 component than that of Iberian Peninsula (Additional file 10: Figure S2). In addition, a high proportion of AA1 component was observed in part of the hspEuropeS strains, similarly to the report about the Portuguese speaking countries by Oleastro et al. [18]. This result suggests that there was gene flow from hpAfrica1 strains brought by African slaves to hpEurope strains brought by the colonial settlers in Central America and Dominican Republic. On the other hand, hspEuropeS strains in Central and South America had significantly higher AEA components than that of the Iberian Peninsula and the Dominican Republic. This suggests that hspEuropeS strains in Central and South America underwent genetic exchange with hspAmerind strains hosted by American aborigines. The ratio of AEA component in the Dominican Republic strains is as low as the strains in the Iberian Peninsula.

These results show that hpEurope strains in the Dominican Republic is different from other countries in that they were highly affected by hpAfrica1 but not by hspAmerind. This country was dominated by colonial settlers first in the American continent, and at the same time, many slaves were forcibly brought from Africa. Therefore, cohabitation history of Europeans and Africans is the longest, and American aborigines might have disappeared in the earliest in the Dominican Republic. Furthermore, genetic exchange with neighboring countries was restricted because of the island environment.

hpAfrica1 strains were divided into three subpopulations: hspWAfrica, hspSAfrica, and hybrid (hspWAfrica/hpEurope). The hybrid (hspWAfrica/hpEurope) strains mainly observed in the country of the Mediterranean coast of the African continent and in the American continent. In the Dominican Republic, pure hspWAfrica and hspSAfrica strains were observed more frequently than other American continent countries. In addition, hybrid (hspWAfrica/hpEurope) strains in the Dominican Republic had significantly less AEA component than that of other American continent countries (Additional file 14: Figure S6). The reason may be the same to the reason of less AEA component in hpEurope strains in the Dominican Republic.

A distinct bacterial population appeared at K = 4 predominant with Nicaragua strains (Additional file 12: Figure S4), which corresponds to hspAfrica1Nicaragua in a previous study [21]. hspAfrica1Nicaragua was reported to be a subpopulation that appeared due to the rapid evolution of the hpAfrica1 strains in American continent. Although a previous study [21] on whole genome data of *H. pylori* reported that there is no evidence that hspAmerind strains contributed DNA to other extraneous New World strains, this study demonstrated that hspAfrica1Nicaragua strains contained high AEA components. This observation supports a hypothesis that the hspAmerind strain disappeared through strain subversion by transformation [22].

The phylogenetic tree showed that hspWAfrica strains in the Dominican Republic were close to the strains in Burkina Faso, Gambia and Senegal (Fig. 4). These countries are located in West Africa where slave trade was thriving. The four hspSAfrica strains in the Dominican Republic that formed a cluster distant from other African strains may derive from a region of African continent with yet no survey of *H. pylori*.

### Relationship between phylogeographical classification of *H. pylori* and host human

The mtDNA is inherited maternally because only mitochondria in the ova are passed on to the child but there is no contribution from the father. Contrary, Y chromosome is inherited paternally to the son. Intra family infection of *H. pylori* occurs often mainly by vertical transmission from a mother who contact to her child intimately. Thus, we initially anticipated that the infection pattern of *H. pylori* might resemble to the inheritance of mtDNA haplogroup rather than Y-haplogroup.

The results that the ratio of hpAfrica1 strains was the highest (57.1%) in *H. pylori* and the ratio of African haplogroup was the highest (80.7%) in mtDNA supported the dominance of mother-to-child infection. However, we observed discrepancy between Amerindian mtDNA and *H. pylori* genotype. Amerindian mtDNA was observed in 11.8% of the individuals but none of them had *H. pylori* that belong to hspAmerind. The reason may be explained by a hypothesis that the adaptability of the hspAmerind strains is lower than that of the hpEurope strains [22, 23]. In addition, a previous study reported that hpAfrica1 strains are as adaptive as hpEurope strains [47]. Therefore, hspAmerind may be the weakest.

The proportion of hpEurope in *H. pylori* (42.0%) was much higher than that of European mtDNA haplogroup (7.6%) because 38.5% of African mtDNA haplogroup and 78.6% of Amerindian mtDNA haplogroup had hpEurope *H. pylori*. These results suggest that infection occurs not only from mother to child but also from father to child, or horizontal transmission occurs from environment such as water supply. However, Amerindian mtDNA haplogroup had significantly higher ratio of hpEurope *H. pylori* and higher amount of European ancestral component (AE1 + AE2) than African mtDNA haplogroup. Thus, the original hspAmerind strains infected to Amerindian aborigines might be replaced by hpEurope strain during the past 500 years. This hypothesis also reflects the history that many aboriginal American women had intermarriage with settler men [65].

In European mtDNA haplogroup, hpAfrica1 *H. pylori* was predominant (6/9 = 66.7%). Although the infection of hpAfrica1 to the patient of European mtDNA could be caused by horizontal transmission, it is also probable that hpAfrica1 might have higher adaptability than hpEurope strains. A previous study predicted that higher genetic diversity is advantageous to adapt the host range [22]. In the Dominican Republic, the genetic diversity of hpEurope was higher than that of hpAfrica1. Thus, genetic diversity does not explain the excess of hpAfrica1 *H. pylori* among individuals of European mtDNA. Further study is needed to clarify the reason.

To our knowledge, this report is the first comparative study between *H. pylori* and mtDNA and Y haplogroups of admixture population in South America. However, there are several limitations in this study. Firstly, the number of male patients was small. For a better understanding of the relationship between Y-haplogroup and *H. pylori*, larger number of male samples will be necessary. Secondly, our samples were taken at a hospital in Santo Domingo, the capital city of the Dominican Republic. The physical and cultural landscape varies by region in the Dominican Republic. Therefore, our results cannot be generalized across the entire region of the Dominican Republic.

## Conclusions

We found that *H. pylori* in the Dominican Republic consists of two populations: hpAfrica1 and hpEurope. Although the Amerindian type of mtDNA haplogroup was observed in 11.8% of the patients, Amerindian type (hspAmerind) of *H. pylori* was not observed. This result supports the hypothesis that hspAmerind strains have lower adaptability than other groups because of low genetic diversity. *H. pylori* strains in the Dominican Republic have different characteristics from South and Central American countries in that they have high component of African ancestry but poor Amerindian component, which reflects the history and the geographic condition of this country.

## Supplementary information

**Additional file 1: Table S1.** Primers used for DNA sequencing of human mitochondrial DNA.

**Additional file 2: Table S2.** Primers used for multiplex amplification of human Y-SNPs.

**Additional file 3: Table S3.** Primers used for minisequencing of Human Y-SNPs.

**Additional file 4: Table S4.** Primers used for DNA sequencing of *H. pylori*.

**Additional file 5: Table S5.** Sources and population assignment of *H. pylori* global reference strains.

**Additional file 6: Table S6.** Results of mitochondrial DNA haplotypes and haplogroups.

**Additional file 7: Table S7.** List of strain information, mitochondrial DNA haplogroup, Y chromosomal haplogroup and population assignment of *H. pylori* in the Dominican Republic.

**Additional file 8: Table S8.** Results of Y chromosomal DNA haplotypes and haplogroups.

**Additional file 9: Figure S1.** Phylogenetic tree of 119 Dominican Republic *H. pylori* strains with 1293 global reference strains.

**Additional file 10: Figure S2.** Box plot diagram of ancestral Africa1 components (AA1) in hspEuropeS subpopulation divided by regions: Iberian Peninsula (*n* = 85), Central America (*n* = 53), South America (*n* = 93), Dominican Republic (*n* = 47). The difference of AA1 ratio between regions was investigated by Kruskal-Wallis test followd by Steel-Dwass post-hoc test.

**Additional file 11: Figure S3.** Box plot diagram of ancestral EastAsia components (AEA) in hspEuropeS subpopulation divided by regions: Iberian Peninsula (*n* = 85), Central America (*n* = 53), South America (*n* = 93), Dominican Republic (*n* = 47). The difference of AEA ratio between regions was investigated by Kruskal-Wallis test followd by Steel-Dwass post-hoc test.

**Additional file 12: Figure S4.** Bayesian subpopulation assignment of 297 hpAfrica1 strains using the no-admixture model (K = 2, K = 3, K = 4) of STRUCTURE software (version 2. 3. 3). 1: Northern Africa (Morocco, Algeria), 2: Western Africa (Cape Verde, Senegal, Gambia, Burkina Faso, Cameroon), 3: Middle Africa (Angola), 4: Eastern Africa (Mozambique, Madagascar), 5: Southern Africa (Namibia, South Africa), 6: Central America (Mexico, Guatemala, Nicaragua, Costa Rica), 7: South America (Colombia, Venezuela, Brazil), 8: Caribbean (Dominican Republic). Colors are coded according to the estimated subpopulation assignment. Each vertical bar represents one sample. The order of the samples is the same in each bar charts.

**Additional file 13: Figure S5.** Box plot diagram of ancestral Europe 1 components (AE1) in the three subpopulations classified by STRUCTURE analysis (no-admixture model, K = 3) of 297 hpAfrica1 strains. The difference of AE1 ratio between subpopulations was investigated by Kruskal-Wallis test followd by Steel-Dwass post-hoc test.

**Additional file 14: Figure S6.** Box plot diagram of ancestral EastAsia components (AEA) in hybrid (hspWAfrica/hpEurope) subpopulation divided by regions: African continent (*n* = 24), Central America (*n* = 29), South America (*n* = 9), Dominican Republic (*n* = 17). The difference of AEA ratio between regions was investigated by Kruskal-Wallis test followd by Steel-Dwass post-hoc test.

**Additional file 15: Figure S7.** Box plot diagram of ancestral EastAsia components (AEA) in the two groups within hybrid (hspWAfrica/hpEurope) subpopulation that indicated by STRUCTURE analysis (no-admixture model, K = 4) of 297 hpAfrica1 strains. The difference of AEA ratio between groups was investigated by Wilcoxon rank sum test.

**Additional file 16: Figure S8.** Box plot diagram of pairwise genetic distances between *H. pylori* strains grouped by bacterial population. The difference of pairwise genetic distance between the bacterial populations was investigated by Wilcoxon rank sum test.

**Additional file 17: Figure S9.** Relationship between phylogeographical classification of *H. pylori* and Y chromosomal haplogroup. (A) Number of *H. pylori* population type in each Y chromosomal haplogroup. Group comparisons were performed using Fisher’s exact test. (B) Box plot diagram of European ancestry components (AE1 + AE2) in each Y chromosomal haplogroup. Group comparisons were performed using Wilcoxon rank sum test.

## Data Availability

Sequencing data for seven housekeeping genes of *H. pylori* and mtDNA of human DNA are available under DDBJ accession numbers LC321074- LC321906 and LC319790- LC320027, respectively.

## Abbreviations

*atpA*: ATP synthase subunit alpha
*efp*: Elongation factor P
*H. pylori*: *Helicobacter pylori*
HV1: Hypervariable Region 1
HV2: Hypervariable Region 2
MLST: Multi Locus Sequence Typing
mtDNA: mitochondrial DNA
*mutY*: A/G-specific adenine glycosylase
*ppa*: Inorganic pyrophosphatase
SNP: Single nucleotide polymorphism
STR: Short Tandem Repeats
*trpC*: Bifunctional indole-3-glycerol phosphate synthase
*ureI*: *Urease accessory protein*
*yphC*: GTP-binding protein
