# Supplementary figures and images for: Comparative study between Helicobacter pylori and host human genetics in the Dominican Republic
Source: BMC Evol Biol. 2019 Nov 1;19:197. doi: 10.1186/s12862-019-1526-9 (PMC6823972; doi:10.1186/s12862-019-1526-9)

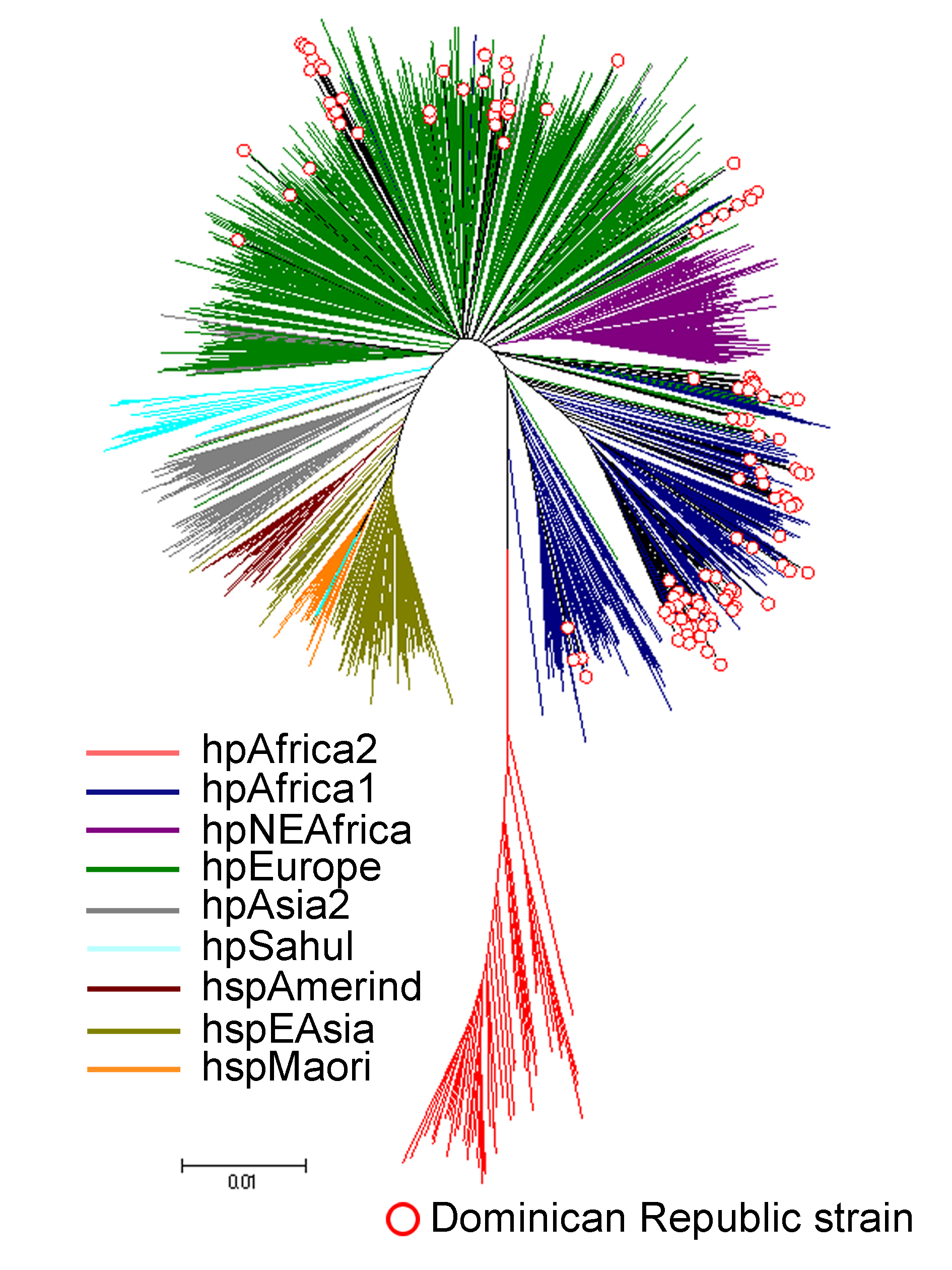

Supplement: Supplementary file 9 — Additional file 9: Figure S1. Phylogenetic tree of 119 Dominican Republic H. pylori strains with 1293 global reference strains. [file 12862_2019_1526_MOESM9_ESM.tif]

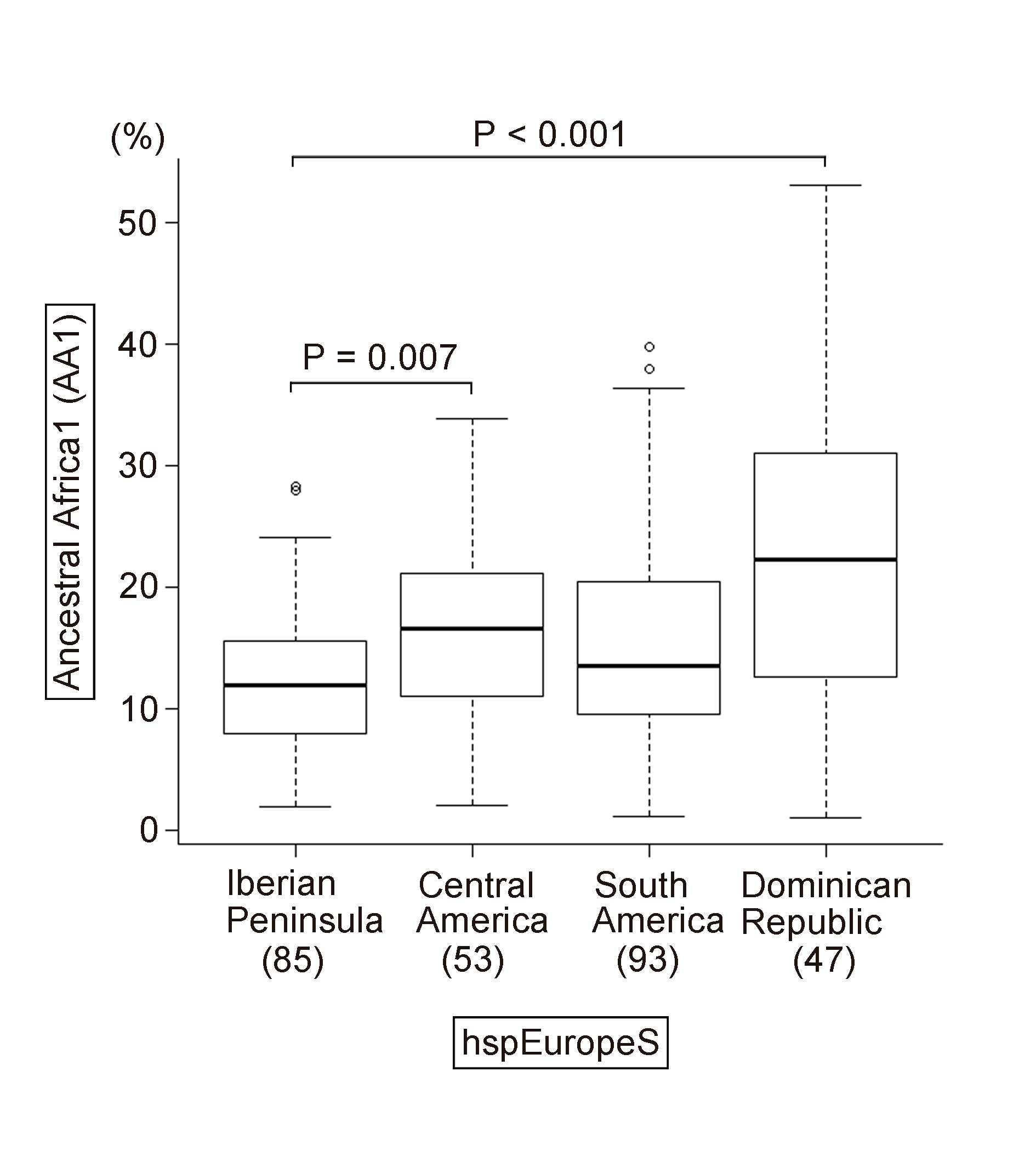

Supplement: Supplementary file 10 — Additional file 10: Figure S2. Box plot diagram of ancestral Africa1 components (AA1) in hspEuropeS subpopulation divided by regions: Iberian Peninsula (n = 85), Central America (n = 53), South America (n = 93), Dominican Republic (n = 47). The difference of AA1 ratio between regions was investigated by Kruskal-Wallis test followd by Steel-Dwass post-hoc test. [file 12862_2019_1526_MOESM10_ESM.tif]

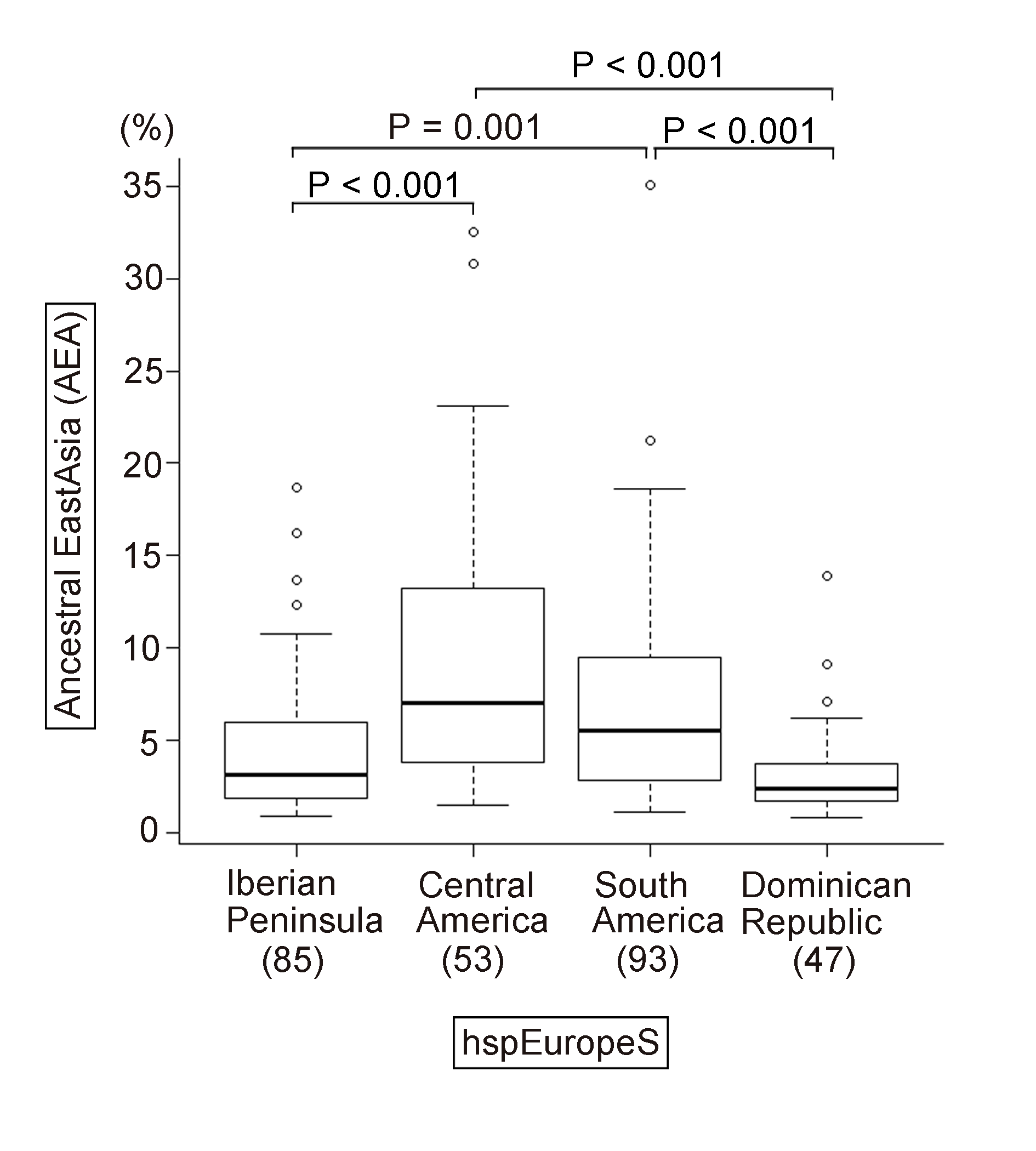

Supplement: Supplementary file 11 — Additional file 11: Figure S3. Box plot diagram of ancestral EastAsia components (AEA) in hspEuropeS subpopulation divided by regions: Iberian Peninsula (n = 85), Central America (n = 53), South America (n = 93), Dominican Republic (n = 47). The difference of AEA ratio between regions was investigated by Kruskal-Wallis test followd by Steel-Dwass post-hoc test. [file 12862_2019_1526_MOESM11_ESM.tif]

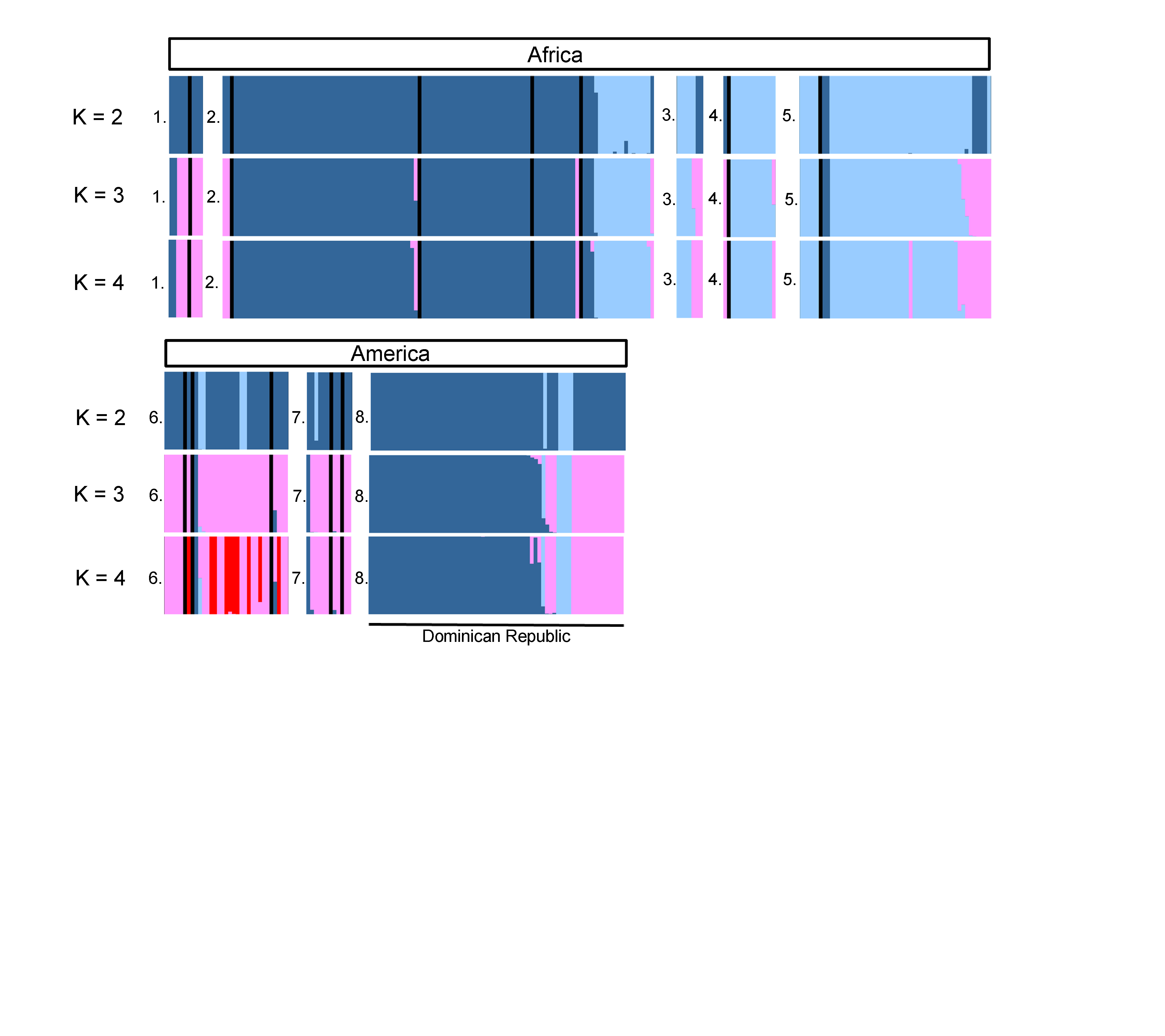

Supplement: Supplementary file 12 — Additional file 12: Figure S4. Bayesian subpopulation assignment of 297 hpAfrica1 strains using the no-admixture model (K = 2, K = 3, K = 4) of STRUCTURE software (version 2. 3. 3). 1: Northern Africa (Morocco, Algeria), 2: Western Africa (Cape Verde, Senegal, Gambia, Burkina Faso, Cameroon), 3: Middle Africa (Angola), 4: Eastern Africa (Mozambique, Madagascar), 5: Southern Africa (Namibia, South Africa), 6: Central America (Mexico, Guatemala, Nicaragua, Costa Rica), 7: South America (Colombia, Venezuela, Brazil), 8: Caribbean (Dominican Republic). Colors are coded according to the estimated subpopulation assignment. Each vertical bar represents one sample. The order of the samples is the same in each bar charts. [file 12862_2019_1526_MOESM12_ESM.tif]

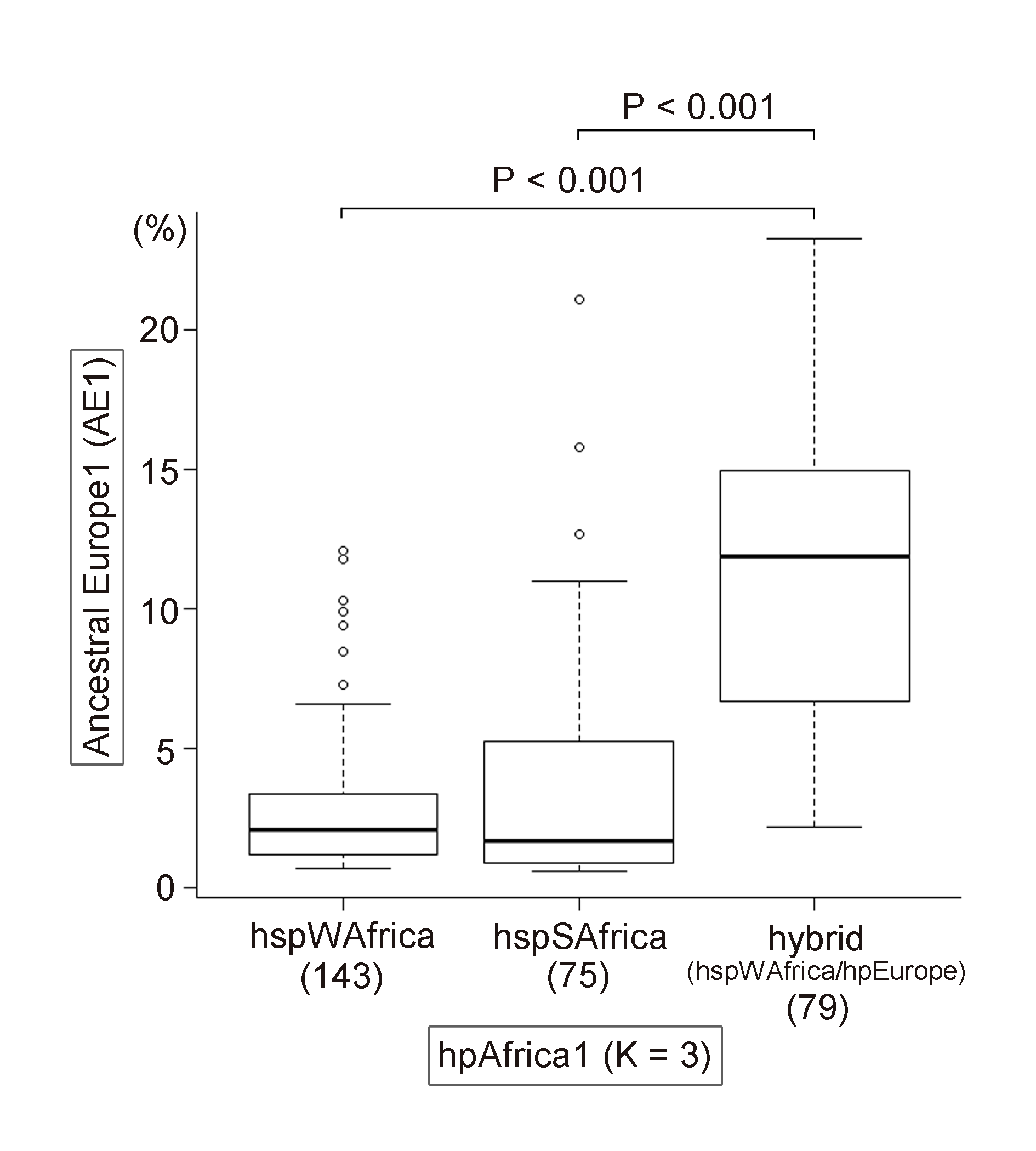

Supplement: Supplementary file 13 — Additional file 13: Figure S5. Box plot diagram of ancestral Europe 1 components (AE1) in the three subpopulations classified by STRUCTURE analysis (no-admixture model, K = 3) of 297 hpAfrica1 strains. The difference of AE1 ratio between subpopulations was investigated by Kruskal-Wallis test followd by Steel-Dwass post-hoc test. [file 12862_2019_1526_MOESM13_ESM.tif]

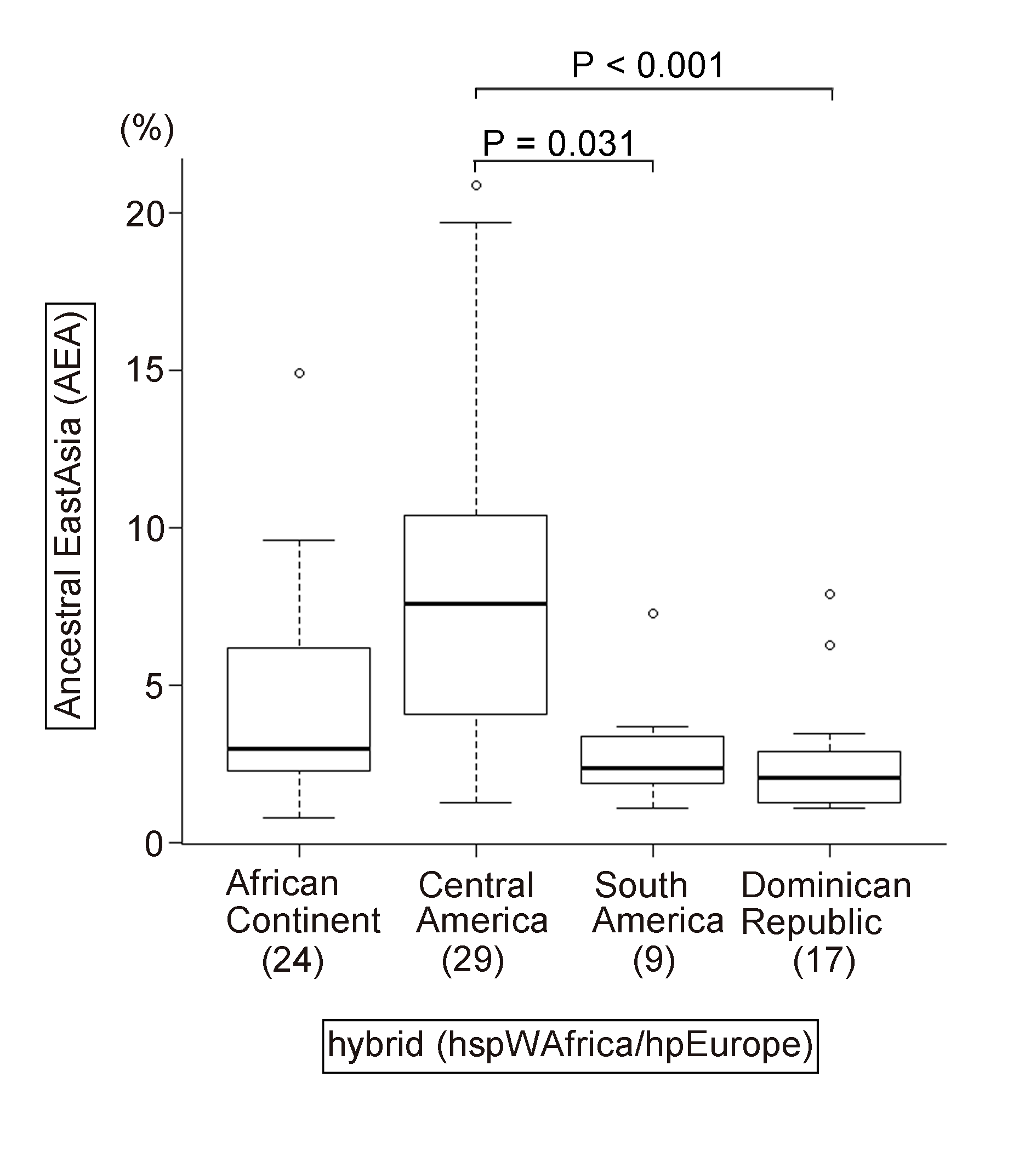

Supplement: Supplementary file 14 — Additional file 14: Figure S6. Box plot diagram of ancestral EastAsia components (AEA) in hybrid (hspWAfrica/hpEurope) subpopulation divided by regions: African continent (n = 24), Central America (n = 29), South America (n = 9), Dominican Republic (n = 17). The difference of AEA ratio between regions was investigated by Kruskal-Wallis test followd by Steel-Dwass post-hoc test. [file 12862_2019_1526_MOESM14_ESM.tif]

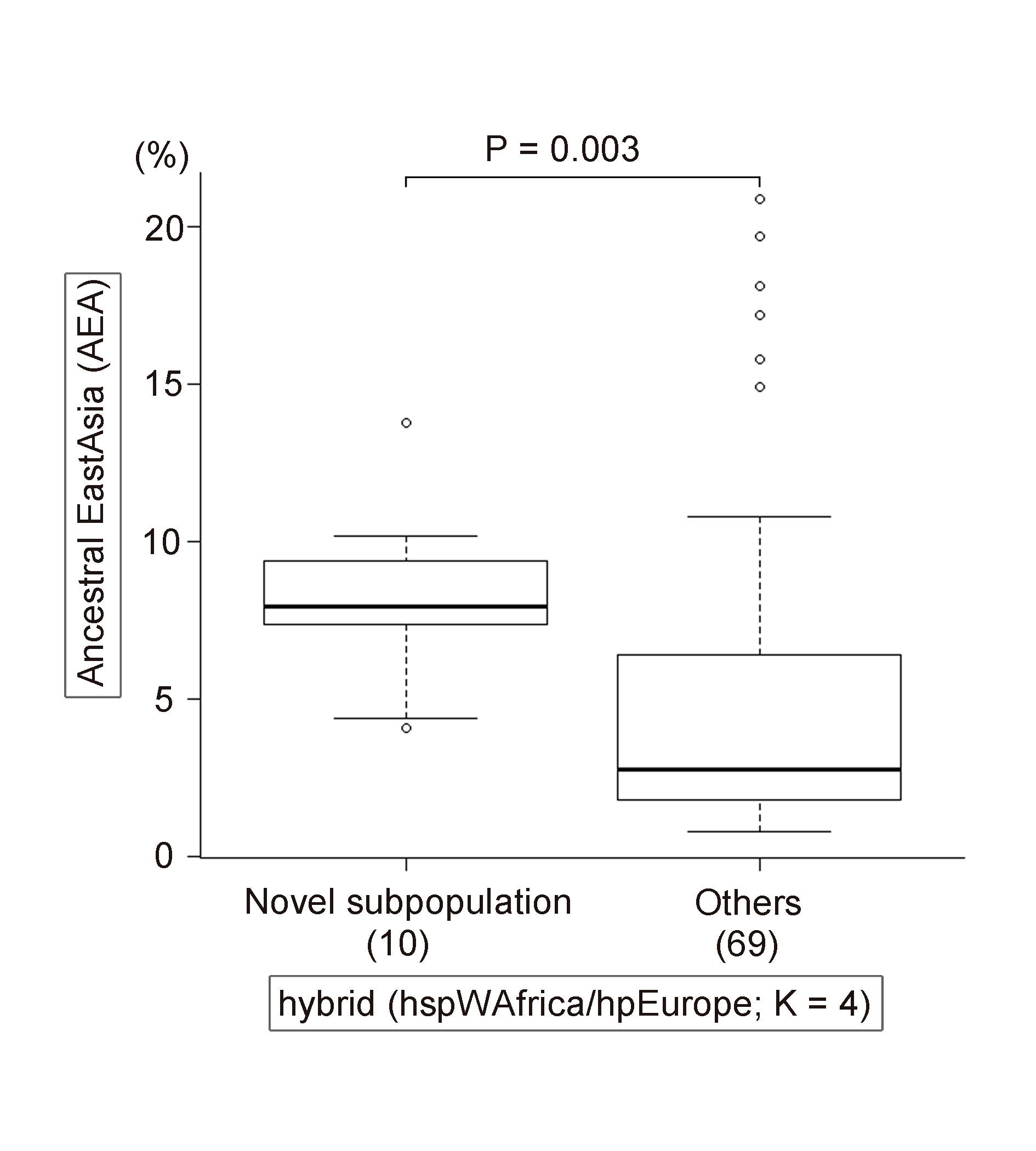

Supplement: Supplementary file 15 — Additional file 15: Figure S7. Box plot diagram of ancestral EastAsia components (AEA) in the two groups within hybrid (hspWAfrica/hpEurope) subpopulation that indicated by STRUCTURE analysis (no-admixture model, K = 4) of 297 hpAfrica1 strains. The difference of AEA ratio between groups was investigated by Wilcoxon rank sum test. [file 12862_2019_1526_MOESM15_ESM.tif]

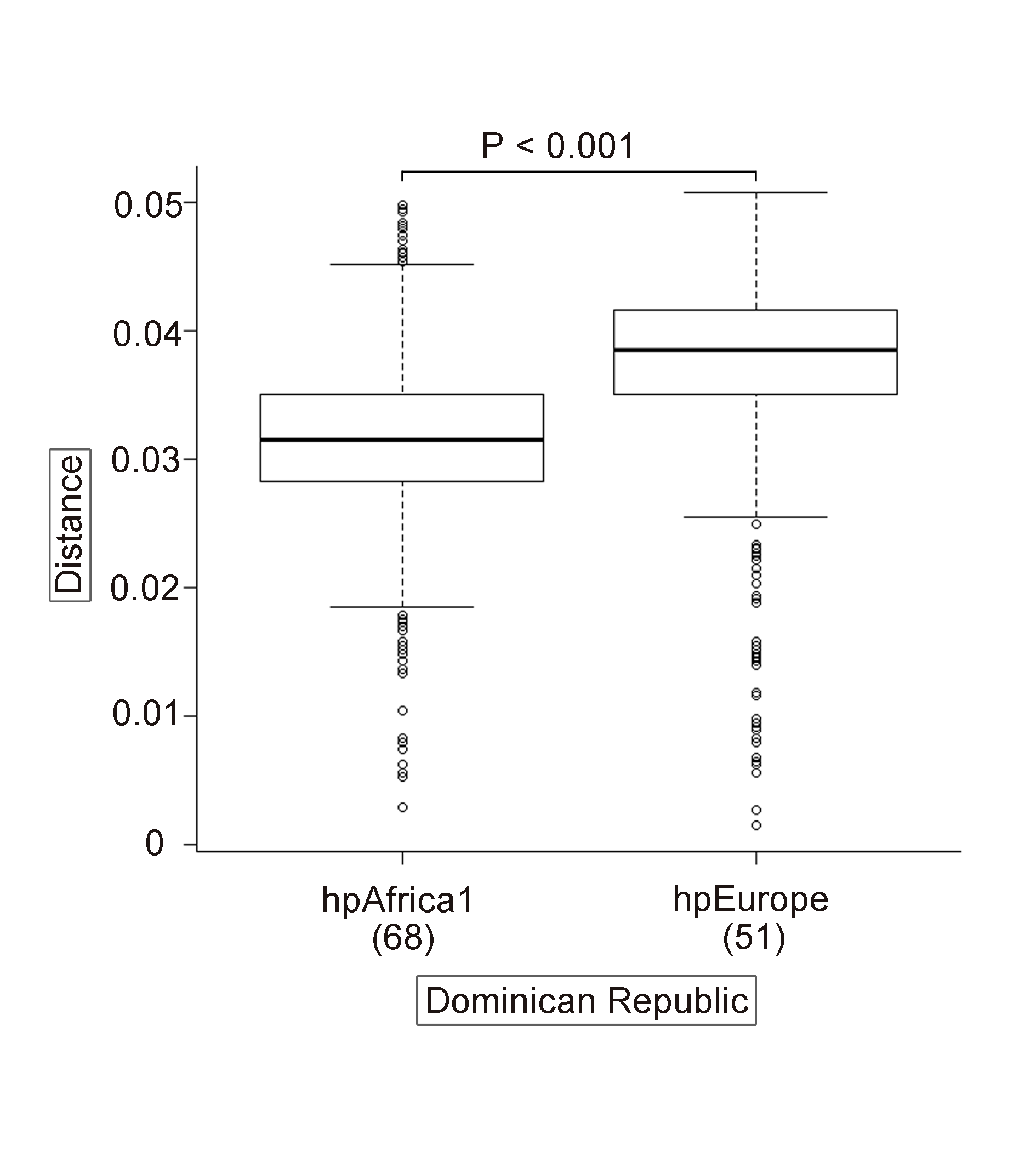

Supplement: Supplementary file 16 — Additional file 16: Figure S8. Box plot diagram of pairwise genetic distances between H. pylori strains grouped by bacterial population. The difference of pairwise genetic distance between the bacterial populations was investigated by Wilcoxon rank sum test. [file 12862_2019_1526_MOESM16_ESM.tif]

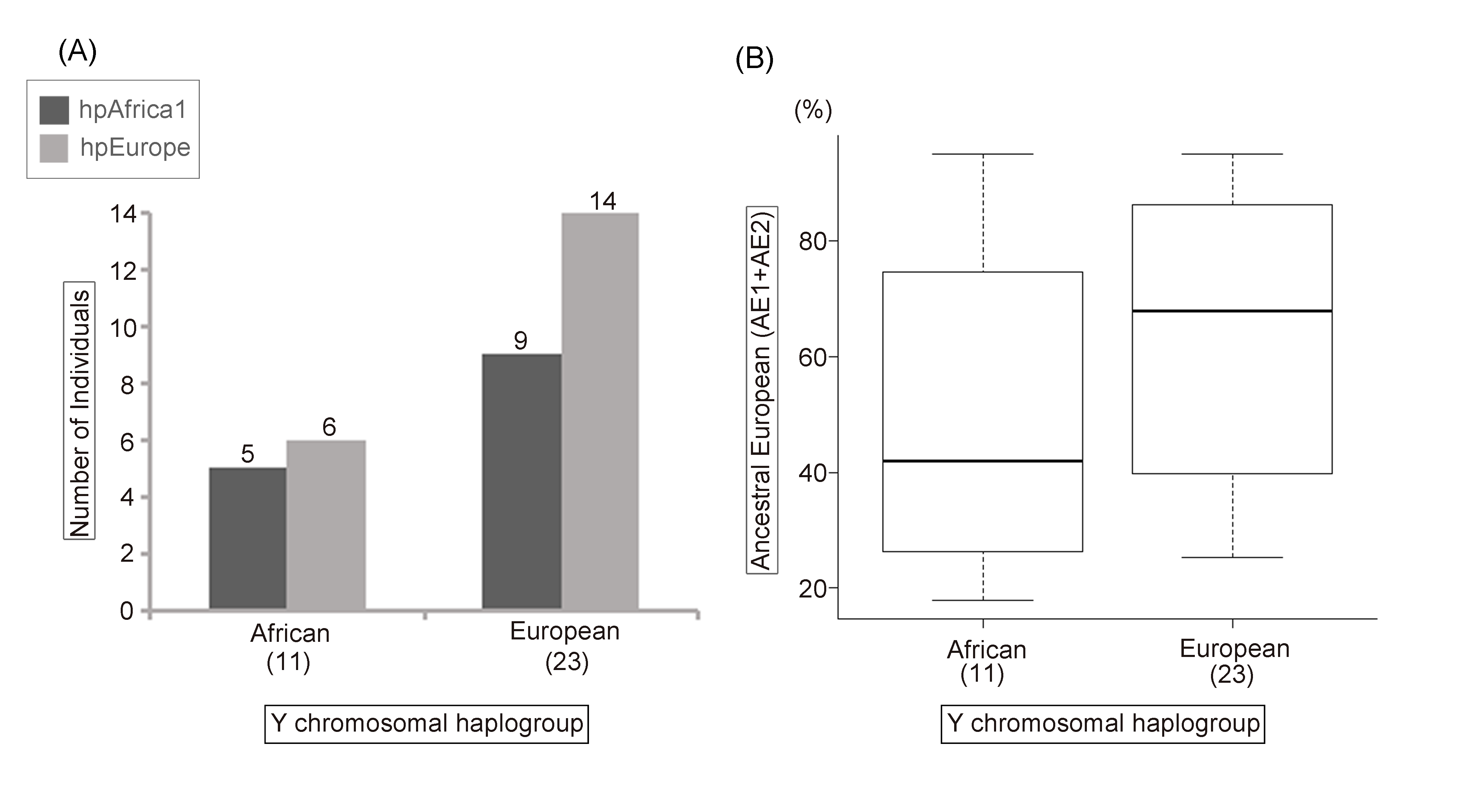

Supplement: Supplementary file 17 — Additional file 17: Figure S9. Relationship between phylogeographical classification of H. pylori and Y chromosomal haplogroup. (A) Number of H. pylori population type in each Y chromosomal haplogroup. Group comparisons were performed using Fisher’s exact test. (B) Box plot diagram of European ancestry components (AE1 + AE2) in each Y chromosomal haplogroup. Group comparisons were performed using Wilcoxon rank sum test. [file 12862_2019_1526_MOESM17_ESM.tif]
